# A Nontoxic Battery with 3D-Printed Housing for On-Demand Operation of Microcontrollers in Microfluidic Sensors

**DOI:** 10.3390/mi10090588

**Published:** 2019-09-04

**Authors:** Kai Sachsenheimer, Christiane Richter, Dorothea Helmer, Frederik Kotz, Bastian Ernst Rapp

**Affiliations:** 1 Laboratory of Process Technology (NeptunLab), Department of Microstructure Engineering (IMTEK), University of Freiburg, Georges-Köhler-Allee 103, 79110 Freiburg, Germany; 2 Freiburger Materialforschungszentrum (FMF), Stefan-Meier-Straße 21, 79104 Freiburg im Breisgau, Germany

**Keywords:** 3D printing, battery, microcontroller, microfluidics, capacitive sensor

## Abstract

Microcontrollers have a low energy consumption and are convenient tools for the operation and readout of small lab-on-a-chip devices. The operation of microcontrollers for data collection and analysis is key for measurements and statistics in field experiments. However, for portable lab-on-a-chip or point-of-care systems in low-resource settings, the availability of energy sources is a bottleneck. Here, we present a simple, nontoxic aluminum/air redox battery with a 3D-printed housing for on-demand operation of a sensor using a microcontroller for data collection. The battery is stored in a dry state and can be manufactured conveniently using off-the-shelf components and a simple 3D printer. It can be quickly assembled and operates a microcontroller for at least one hour in continuous operation mode. We demonstrate its performance by collecting data from a capacitive sensor capable of determining the conductivity of liquid samples. Such sensors can be used for, e.g., determining the water quality or phase formation in liquid mixtures. The sensor performance in determining different conductivities of nonconductive and conductive liquids in droplets is demonstrated.

## 1. Introduction

The field of “mobile sensing” has rapidly evolved in the past years. As early as 2008, mobile phones were used for collecting data of microfluidic assays [1]. The field of microfluidic sensing based on smartphones, sometimes abbreviated as MS^2^, has come a long way, and a number of different tests have been implemented, including fluorescence [2,3], Mie scattering [4], and loop-mediated isothermal amplification (LAMP) [5,6]. Using a smartphone, the power source and data collection are directly combined. The use of smartphones comes with two major drawbacks. Firstly, the readout methodology with smartphones is usually based on image analysis, which is generally not a particularly sensitive method, often generating merely a “yes” or “no” answer and requiring labeling of the analyte. For more sensitive, label-free readouts, electrical sensors are preferred [7]. Secondly, smartphones are not convenient for low-resource settings which lack energy sources, since they are costly and require frequent recharging. To create an independent device, a battery-powered microcontroller that collects data for USB readout via a RS-232 interface is a low-cost, robust alternative. Microcontrollers have been identified as key enablers for the next generation of lab-on-a-chip (LOC) devices and point-of-care (POC) diagnostics [8]. The separation of the data collection from the energy source ensures a much smaller energy consumption than, e.g., smartphone-based image analysis methods. Microcontrollers have a low energy consumption and thus can be operated by simple batteries. 

Compared to fuel cells, which require the storage of hydrogen, batteries are convenient, low-cost, portable energy sources that are simple to use and easy to set up. To meet the requirements for low-resource settings, batteries must be easy to produce, cheap, long-lived, and disposable. Disposability is especially critical for batteries based on very reactive elements such as lithium. Among the many different batteries, batteries for “on-demand” operation are of special interest since their shelf life can be greatly increased [9]: In an on-demand device, the electrolyte is added just prior to the operation, which keeps the battery from self-discharging during storage. Several on-demand batteries have been reported, based on different battery types. Lee et al. described a micro battery consisting of Au (gold) and Zn (zinc) electrodes fabricated with silicon micromachining [10]. The electrodes are separated and the battery is activated by adding the liquid electrolyte (sulfuric acid). A similar approach with Mg (magnesium) as the anode, AgCl (silver chloride) or CuCl (copper chloride) as the cathode, and water as the electrolyte was demonstrated by Sammoura et al. [11]. Thom et al. presented a galvanic cell inside a microfluidic channel which was activated by adding the probe and demonstrated its functionality in a UV-LED-based fluorescence assay on-chip [12]. 

In terms of basic battery setups, metal/air batteries offer several advantages: they combine high energy density and capacity; the storage capacity is independent of charge and temperature; and they have low production costs, low discharge voltages, long shelf life at dry storage, and can be assembled from environmental harmless materials, which is why they are studied as alternative energy storage systems [13]. They do not require highly reactive metals or hazardous elements such as mercury or cadmium [14]. Compared to lithium-ion batteries, metal-air batteries exhibit 2–10 fold higher energy densities [15]. Aluminum/air batteries combine the advantages of light weight, high energy density, low cost, and easy disposal. Several on-demand aluminum/air batteries have been reported. Cardenas-Valencia et al. presented an aluminum/air battery made via silicon micromachining which can be activated via microfluidics on demand. Activation is induced by thermal expansion of a working fluid [16] or pneumatically as a result of electrolysis [9]. Combining the concept of 3D printing with the use of nontoxic, readily available materials to create a disposable power source enables the quick and easy fabrication of reliable batteries that can be used to power sensor devices in low-resource settings. 

In this paper, we present a custom-designed aluminum/air battery developed as a stand-alone power supply which can be manufactured via 3D printing. The battery contains no toxic components and is activated by adding the electrolyte. We demonstrate the suitability of this device for powering a sensor system capable of measuring the conductivity of liquids. The sensor system is operated by a microprocessor with peripheral electronics mounted on a single printed circuit board (PCB) onto which the 3D printed battery is plugged.

## 2. Materials and Methods 

**Figure 1:**
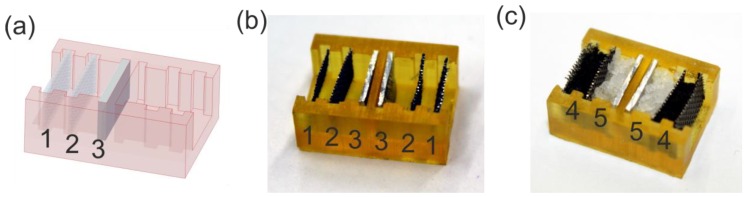
3D-printed battery with distinct electrolyte chambers. (**a**) Battery design showing two individual battery parts with two platinum-coated meshes (1, 2) and one aluminum sheet (3). (**b**) Printed battery with platinum meshes (1, 2) and aluminum sheets (3). (**c**) Fully assembled battery from (**b**) with additional charcoal (4) and superabsorber/potassium hydroxide filling (5).

**Battery manufacturing**: A battery housing was fabricated using a commercial stereolithography system (Asiga Pico 2, 3DXS, Erfurt, Germany) using the resin luxaprint 3D mold (purchased from DETAX GmbH & Co. KG, Ettlingen, Germany). Two stainless steel meshes (size: 10 × 5 mm², thread diameter: 195 µm, mesh size: 500 µm) were sputtered with a 100 nm Pt layer. This mesh and a piece of 10 × 5 mm² aluminum sheet (thickness: 1 mm) were inserted into the housing as shown in Figure 1. The chamber between the two meshes was filled with active charcoal (grain size: 0.3–0.5 mm, purchased from Merck, Darmstadt, Germany) serving as the air electrode. The second chamber intended for the electrolyte was covered with a thin layer of a superabsorbent polymer, here 12 mg poly(acrylic acid), taken from a baby diaper. Before usage of the battery, the electrolyte (200 µL 3 M KOH solution) was added onto the superabsorbent polymer.

**Sensor manufacturing**: A PCB board was designed and printed for the capacitive sensor, which includes two sensing electrodes. The sensing electrodes were passivated by a layer of parylene C (1 µm, deposited via chemical vapor deposition) to avoid corrosion and electrolysis at the electrodes as well as faradaic current flow. The battery housing was glued onto the PCB with an epoxy-based glue (UHU® plus endfest, UHU, Bühl, Germany) and a conductive adhesive (CircuitWorks® Conductive epoxy, Chemtronics, Kennesaw, GA, USA). 

**Manufacturing of microfluidic channel for fluid sensing**: A microfluidic device was fabricated via soft lithography from a template master made via stereolithography from luxaprint 3D resin. A two-component polydimethylsiloxane (PDMS, type Elastosil RT 601, purchased from Wacker Chemie, Munich, Germany) was mixed in the mass ratio 9:1 (component A:B), degassed, and cast against this master structure and cured to generate the sensing channel. A frame was 3D-printed and used to press the PDMS chip to the PCB board using screws. In this way, a channel was created on top of the sensor electrodes. Teflon tubing was connected by plugging the tubing directly into the chip.

**Sensing of fluid conductivity:** To test the performance, operation time, and response time of the battery-powered sensor, alternating droplets of conductive and nonconductive fluids were passed over the sensing electrodes. A nonconductive fluid (FC-40, Sigma Aldrich, Taufkirchen, Germany) and a conductive fluid (water, 300 µS/cm) were introduced at the two inlets of a T-junction microfluidic channel. The flow was driven by syringe pumps (type Legato® 110, KD Scientific, Holliston, MA, USA) and adjusted to 20 µL/min for both fluids. The individual droplets formed were used to collect conductivity data of both fluids. 

## 3. Results

### 3.1. 3D-Printed Battery

A battery was designed with the goal of generating a simple, nontoxic, and disposable on-demand power supply capable of operating a microcontroller independently. For this purpose, a battery housing was 3D-printed using a commercial benchtop STL printer (see Figure 1). The individual components of the battery (platinum-coated steel mesh, aluminum sheets, charcoal, and superabsorber) were added to this housing. Just before use, potassium hydroxide was introduced as an electrolyte, swelling the superabsorber, thus generating a gel-like electrolyte phase (see Figure 1c). 

**Figure 2:**
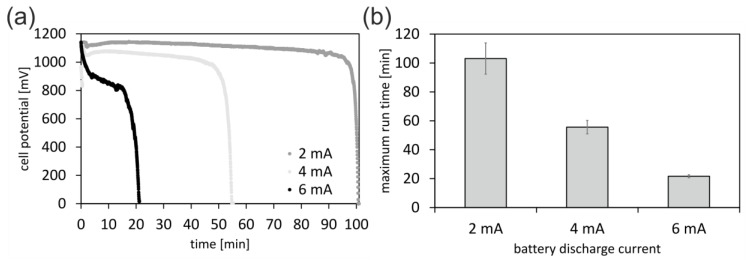
Battery performance analysis. (**a**) The discharge behavior of the battery was tested at discharge currents of 2 mA, 4 mA, and 6 mA. At lower discharge currents, the cell potential remains largely constant and only drops dramatically when the electrolyte is completely consumed. (**b**) At discharge currents of 2 mA, 4 mA, and 6 mA, the battery operates for an average of 100 min, 60 min, and 20 min, respectively.

We first characterized the performance of the battery by measuring the discharge behavior at different constant discharge currents of 2 mA, 4 mA, and 6 mA. The theoretical cell voltage of an aluminum/air battery using an alkaline electrolyte is 2.7 V, but due to oxidation of the aluminum and parasitic corrosion, the achievable operating values are between 1.2 V and 1.6 V [17]. In simple battery setups, the cell potential is reduced due to a parasitic reaction at the aluminum/water interface [18], and the maximum cell potential of the 3D-printed battery was measured at 0 s discharge time and determined to be 1.1 ± 0.1 V. For all measured discharge currents, the cell potential was stable and allowed prolonged and constant operation. At a discharge current of 2 mA, the battery could be operated for an average of 100 min (see Figure 2). Upon complete consumption of the alkaline electrolyte, the cell potential drops. The initial drop-off for 6 mA can be explained by the charge mobility in the electrolyte as well as at the interface. This dynamic effect is less pronounced at lower currents, where diffusion is fast enough for delivering a constant output voltage. The mean nominal capacity of a battery with a constant discharge current of 2 mA and a discharge up to 70% of the maximum cell potential was 12.2 ± 1.0 As. The nominal capacity decreases with higher discharge currents, because the voltage drop at the internal resistance of the cell increases and the mobility of the charge carriers within and on the boundary of the electrodes is reduced. The power output of the battery was determined to be 0.4 mW for one hour or 0.2 mW for two hours.

### 3.2. Portable Conductivity Sensor with Microcontroller

**Figure 3:**
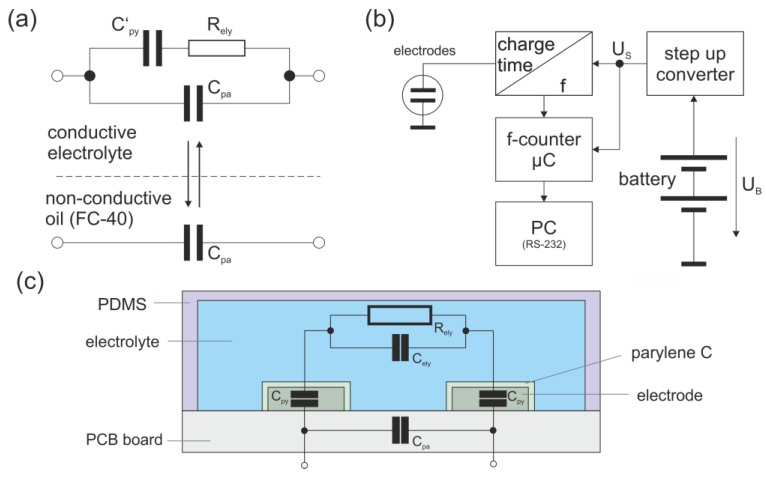
Equivalent circuit diagram and setup of the conductivity sensor. (**a**) Simplified equivalent circuit of the sensor setup. Alternating contact with conductive and nonconductive liquids changes the electrical properties of the electrodes. *C_pa_* includes all parasitic capacities, such as stray capacitance of the printed circuit board (PCB), input capacitance of the electronic system, etc. *C’_py_* is the capacity of the insulating parylene C layer above the electrodes, and *R_ely_* represents the ohmic resistance due to the limited conductibility of the electrolyte. (**b**) Block diagram of the electronic system used for sensing of the conductivity. A step-up converter upregulates the battery voltage *U_B_* to the supply voltage *U_S_* required for the microprocessor. The charge and discharge times of the capacitor are monitored and converted into a rectangular signal with corresponding frequency. The frequency is read out via a microcontroller and communicated to a PC via a RS-232-interface. (**c**) Scheme of the setup of the microfluidic channel with the sensing electrodes.

To generate a portable sensor, a PCB board for conductivity measurements was designed (see Figure 3). The setup contains a microcontroller for data collection and passivated electrodes in a microfluidic channel for the conductivity measurements. The two electrodes are operating as a capacitive circuit: when the electrodes are probed with liquids of different conductivity, the electrical characteristic changes periodically. The custom-made aluminum/air battery supplies the voltage *U_B_*, which is upregulated to the supply voltage *U_S_* required for the microprocessor by means of a step-up converter. To achieve *U_S_* = 2.8 V, a minimum *U_B_* of 0.8 V is required. The required current of the circuit is 2.1 mA. The internal capacitance of the sensor electrodes is charged up to 2/3∙*U_S_* via a charging resistor and discharged to 1/3∙*U_S_* via a discharge resistor. This procedure is repeated continually. The charge and discharge times were converted to a rectangular signal with the appropriate frequency. This frequency is read out by a microcontroller (ATTINY 861A-SU, Atmel, San Jose, CA, USA) and communicated to a PC via a RS-232-interface. With a conductive liquid, the capacity of the circuit is increased, resulting in an increasing charging time and therefore decreasing frequency. The circuit is dimensioned to sense fluids with a conductivity between 50 µS/cm and 400 µS/cm with a counting rate up to 50 Hz. When probed with conductive electrolytes such as water, the circuit contains the parasitic capacitance with a parallel branch containing the double-layer capacitance of the parylene C layer, which passivates the electrodes, in series with an ohmic resistor representing the electrolyte conductivity. If a nonconductive liquid is probed across the electrodes, only the parasitic capacitance is relevant. The system measures the changing charging and discharging characteristics as a consequence of the segmented flow. 

**Figure 4:**
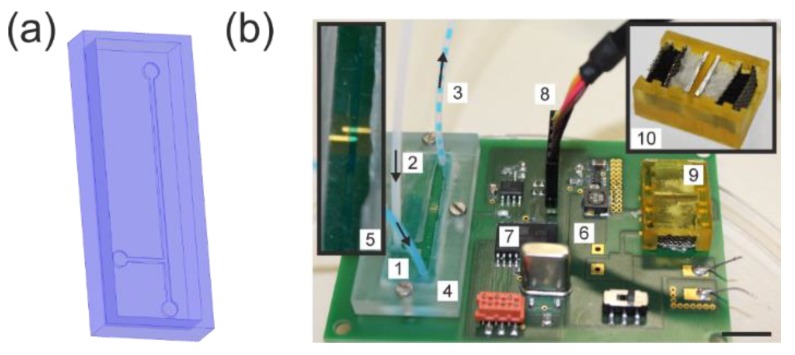
Complete sensor device with 3D-printed microfluidic channel setup. (**a**) A T-junction for the creation of alternating fluid droplets was created in polydimethylsiloxane (PDMS) and attached to the PCB board via a 3D-printed frame. (**b**) View of the assembled sensor platform consisting of a printed circuit board (PCB), microfluidic structure, and the custom-made aluminum/air battery which powers the system: 1—inlet flow (aqueous phase with blue dye), 2—inlet flow (FC-40 phase), 3—outlet segmented flow, 4—3D-printed holding frame with inserted PDMS microfluidic chip (shown in **a**), 5—detailed view of the microchannel with the two sensing electrodes, 6—PCB, 7—microprocessor, 8—connection to PC via RS232 interface, 9—custom-made aluminum/air battery which powers the entire system, 10—detailed view of the battery. Scale bar: 1 cm.

To allow for a controlled contact of the sensing electrodes and the fluid, a rectangular microfluidic channel of 500 µm × 500 µm was placed on top of the passivated electrodes (see Figure 4a). The channel was fixed to the device by a 3D-printed frame screwed to the PCB board. A T-junction was chosen to allow for generation of droplets to demonstrate the detection of alternating fluids in real time. Alternating droplets of water (colored with blue ink) and FC-40 were probed across the electrodes. Just before use, the battery was assembled and attached to the board for operating the microcontroller. The complete device is shown in Figure 4b.

### 3.3. Conductivity Sensor Data Collection with Battery-Operated Microcontroller

**Figure 5:**
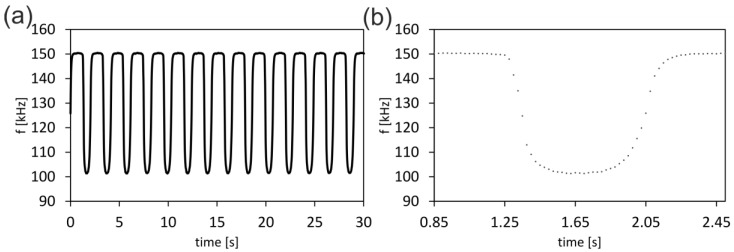
Sensor performance and response time. (**a**) Alternating droplets of conducting fluid (water) and nonconducting fluid (FC-40) were passed over the sensing electrodes. The electrodes detected the change in conductivity, resulting in a change of the charge and discharge time of the capacitor, which is displayed as a change in the corresponding frequency. The battery-operated microcontroller ensures a quick and stable readout of the alternating conductivity over 100 min at 2 mA discharge current. (**b**) Sensor response time: Close-up of the detected frequency change. The sensor detects the change between the two fluids in less than 0.4 s.

To test the performance of the battery upon field use of the conductivity sensor and to determine its response time, fluids of alternating conductivity were passed through the microfluidic setup containing the electrodes. Droplets of distilled water were adjusted to the conductivity of regular tap water by introducing potassium salts to achieve a conductivity of 300 µS/cm. FC-40 fluorinated hydrocarbon was used as a nonconductive fluid. The sensor was capable of detecting the change of conductivity between the two fluids in less than 0.4 s (see Figure 5). With one complete battery consisting of two cells, the sensor could be operated for at least one hour. 

## 4. Conclusions

Aluminum/air batteries are reliable, easy-to-handle power sources with a high energy density. For in-field use, especially in low-resource settings, these easy-to-set-up, disposable, nontoxic batteries with long shelf life can be key enablers for on-site monitoring and sensing applications. In this work, we demonstrated such a battery which can be assembled from affordable, off-the-shelf components mounted into a 3D-printed housing. As an exemplary application, a capacitive sensor was designed for the detection of fluid conductivity using a state-of-the-art microcontroller. The low energy consumption of modern microcontrollers and the label-free and sensitive detection of electrical sensors ensures versatile use of the system. The battery reliably operates the device for 100 min at a discharge current of 2 mA. This portable sensor setup is capable of detecting fluid conductivity in real time and is thus an interesting tool for monitoring fluid quality with low energy consumption.
